# Genetic and transcriptomic analysis of transcription factor genes in the model halophilic Archaeon: coordinate action of TbpD and TfbA

**DOI:** 10.1186/1471-2156-8-61

**Published:** 2007-09-24

**Authors:** James A Coker, Shiladitya DasSarma

**Affiliations:** 1 University of Maryland Biotechnology Institute, Center of Marine Biotechnology, 701 East Pratt Street, Baltimore, MD 21202, USA

## Abstract

**Background:**

Archaea are prokaryotic organisms with simplified versions of eukaryotic transcription systems. Genes coding for the general transcription factors TBP and TFB are present in multiple copies in several Archaea, including *Halobacterium *sp. NRC-1. Multiple TBP and TFBs have been proposed to participate in transcription of genes via recognition and recruitment of RNA polymerase to different classes of promoters.

**Results:**

We attempted to knock out all six TBP and seven TFB genes in *Halobacterium *sp. NRC-1 using the *ura*3-based gene deletion system. Knockouts were obtained for six out of thirteen genes, *tbp*CDF and *tfb*ACG, indicating that they are not essential for cell viability under standard conditions. Screening of a population of 1,000 candidate mutants showed that genes which did not yield mutants contained less that 0.1% knockouts, strongly suggesting that they are essential. The transcriptomes of two mutants, Δ*tbp*D and Δ*tfb*A, were compared to the parental strain and showed coordinate down regulation of many genes. Over 500 out of 2,677 total genes were regulated in the Δ*tbp*D and Δ*tfb*A mutants with 363 regulated in both, indicating that over 10% of genes in both strains require the action of both TbpD and TfbA for normal transcription. Culturing studies on the Δ*tbp*D and Δ*tfb*A mutant strains showed them to grow more slowly than the wild-type at an elevated temperature, 49°C, and they showed reduced viability at 56°C, suggesting TbpD and TfbA are involved in the heat shock response. Alignment of TBP and TFB protein sequences suggested the expansion of the TBP gene family, especially in *Halobacterium *sp. NRC-1, and TFB gene family in representatives of five different genera of haloarchaea in which genome sequences are available.

**Conclusion:**

Six of thirteen TBP and TFB genes of *Halobacterium *sp. NRC-1 are non-essential under standard growth conditions. TbpD and TfbA coordinate the expression of over 10% of the genes in the NRC-1 genome. The Δ*tbp*D and Δ*tfb*A mutant strains are temperature sensitive, possibly as a result of down regulation of heat shock genes. Sequence alignments suggest the existence of several families of TBP and TFB transcription factors in *Halobacterium *which may function in transcription of different classes of genes.

## Background

Archaea drive transcription using a simplified version of a eukaryotic RNA polymerase II-like transcription system made up of 11–13 subunits [1-5]. The first step in transcription requires the binding of the TATA-binding protein (TBP) transcription factor to the TATA box, which results in bending of the DNA [6]. When the TBP-DNA complex is correctly oriented, it is bound by TFB (homolog of eukaryotic TFIIB) to a region just upstream of the TATA box (B recognition element) and is responsible for the polarity of transcription. This contrasts with eukaryotic pol II promoters which are bound by additional TBP-associated factors (TAFs). Finally, RNA polymerase is recruited and transcription is initiated [4,7-9]. We sought to understand the process of transcription in halophilic archaea, using the model halophilic archaeon, *Halobacterium *sp. NRC-1.

The general model for transcription in archaea was complicated by the discovery of a multiplicity of TBP (six) and TFB (seven) genes in the genome sequence of *Halobacterium *sp. NRC-1 [10,11]. The inventory of transcription genes in NRC-1 included the RNA polymerase, coded by twelve genes located at 6 loci [12]. Genes encoding Rpo subunits A, C, B', B", and H [13], and subunits E' and E", as well as subunits K, N, and M, were all found in single copies on the 2 Mbp chromosome. The transcription factors genes, in contrast, were present on both the chromosome and pNRC100 and pNRC200 minichromosomes (which are 191 and 365 kbp, respectively, in size), including four *tbp *genes on pNRC100 (*tbp*A, B, C, and D) and additional single genes on both pNRC200 (*tbp*F) and chromosome (*tbp*E) [12,14]. Five of the seven *tfb *genes were present on the large chromosome (*tfb*A, B, D, F, and G) and the other two were on pNRC200 (*tfb*C and E). These results suggested the possibility of a novel regulatory system involving the recognition of different classes of promoters by specific TBP-TFB combinations [10,15,16].

The possibility of multiple classes of promoters recognized by different factors was supported by promoter mutagenesis studies. These studies showed that while some *Halobacterium *genes have the requirement of a canonical TATA box [17], others deviate from the consensus [11]. One of the best studied examples, the bacterio-opsin (*bop*) gene promoter, contains a TATA-box (GTTACA) [11,18] which deviates significantly from the archaeal consensus (TTATA). Moreover, in the region immediately 5' to the *bop *TATA-box, there is no recognizable BRE sequence functioning in TFB recognition. These results suggest the requirement of a non-canonical TBP-TFB pair in promoter selection and transcriptional regulation for some haloarchaeal gene promoters. These findings suggest unusual complexity in promoter selection in halophilic archaea, which may in some respects resemble higher organisms [10,11,19-22].

In addition to transcription, a variety of genetic characteristics of *Halobacterium *sp. NRC-1 have been studied in our laboratory and other laboratories [23]. The popularity of NRC-1 for post-genomic studies stems from its ease of manipulation in the laboratory and the wide variety of experimental tools available for its investigation [24,25]. Culturing of this model organism, which has a 6 hour generation time at 42°C, is simple and it is genetically tractable. *Halobacterium *sp. NRC-1 is transformable at high-efficiency, and a good selection of cloning and expression vectors are available [26-29]. Several genetic markers have been developed, including the selectable and counterselectable *ura*3 gene, which permit construction of systematic gene knockouts and replacements [30-38]. Whole-genome DNA microarrays have been successfully used to interrogate patterns of gene expression [39-43].

In the present study, we addressed the role of specific TBP-TFB pairs in promoter selection by a combination of genetic and transcriptomic analysis. We found nearly half of the *tbp *and *tfb *genes are non-essential and may be knocked-out without deleterious effects on the cells grown under standard laboratory conditions. Comparison of two of these gene deletion strains, Δ*tbp*D and Δ*tfb*A, showed a high correspondence in the genes that apparently require them for transcription as well as a reduced growth rate and viability at elevated temperature, consistent with reduced transcription of heat shock protein genes.

## Results

### Construction of *tbp *and *tfb *knockout strains

**Figure 1:**
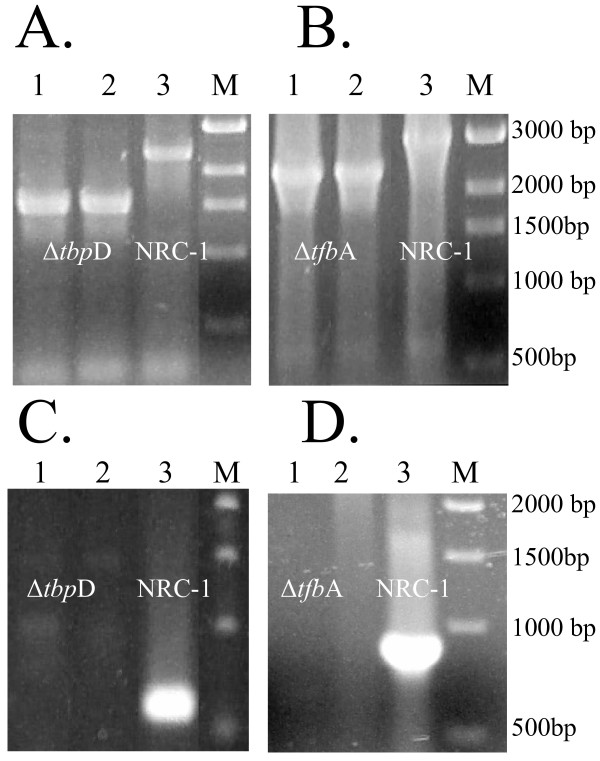
Agarose gel electrophoresis of PCR amplification targeting the flanking (A, B) and exact (C, D) gene regions of *tbp*D (A, C) and *tfb*A (B, D) in the knockout strains. In all panels, lanes 1 and 2 are with genomic DNA from two independently derived knockout strains (Δ*tbp*D or Δ*tfb*A), lane 3 is with genomic DNA of NRC-1, and lane M is a marker (sizes are noted to the right).

**Figure 2:**
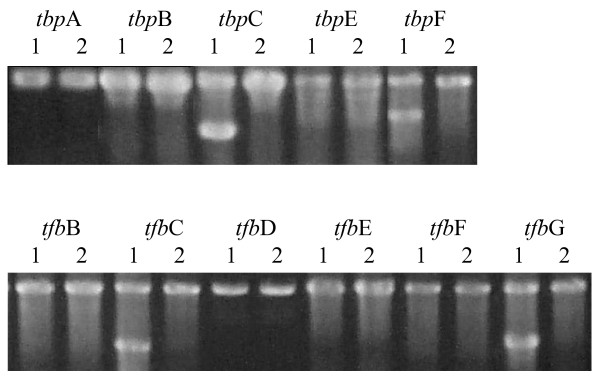
Agarose gel electrophoresis of PCR amplified fragments of the area surrounding the *tbp *and *tfb *genes of *Halobacterium *sp. NRC-1. Template DNA used in Lane 1 of each set is genomic DNA from a population of deletant candidates (roughly 1000 colonies grown on CM^+^-5FOA agar). Template DNA used in Lane 2 is genomic DNA from the parental strain. Lanes with a higher and lower band are representing the wild-type and knockout allele, showing the transcription factor is non-essential. For the *tbp *genes the knockout allele is roughly 500 bp below the wild-type allele for the *tfb *genes it is roughly 900 bp below.

To determine the essentiality of the six *tbp *and seven *tfb *genes in *Halobacterium *sp. NRC-1 and study their involvement in gene expression, we attempted to individually knock out all 13 genes using the Δ*ura*3 system [26]. For this work, ~500 bp of the 5' and 3' flanking sequence and the first four and last four codons of each of the 13 *tbp *and *tfb *genes were cloned into the pBB400 or pMPK408 suicide vector (see additional files 3 and 4), and transformed into the Δ*ura*3 mutant of wild-type *Halobacterium *sp. NRC-1. Integrant strains were selected on uracil dropout media and excisants were selected on agar plates containing 5-fluoroorotic acid (5-FOA) [31,38,42]. PCR amplification was conducted on DNA template from 20 to 70 Foa^R ^colonies using flanking primers to screen for each gene deletion. Knockouts were obtained for six genes: *tbp*C, *tbp*D, *tbp*F, *tfb*A, *tfb*C, and *tfb*G, indicating that these genes are non-essential under standard laboratory growth conditions (Figures 1 and 2, Table 1).

**Table 1:** List of strains

Strain	Phenotype	Genotype	Microarray results
Δ*ura*3	5FOA sensitive	Δ*ura*3	
Δ*tbp*C	None apparent	Δ*ura*3Δ*tbp*C	N.D.^a^
Δ*tbp*D	Heat sensitive growth	Δ*ura*3Δ*tbp*D	413 genes regulated
Δ*tbp*F	None apparent	Δ*ura*3Δ*tbp*F	N.D.^a^
Δ*tfb*A	Heat sensitive growth	Δ*ura*3Δ*tfb*A	482 genes regulated
Δ*tfb*C	None apparent	Δ*ura*3Δ*tfb*C	N.D.^a^
Δ*tfb*G	None apparent	Δ*ura*3Δ*tfb*G	N.D.^a^

^a^Not determined

For the remaining seven genes, for which deletants were not obtained, we assayed for the presence of knockout alleles using populations of 1,000 Foa^R ^colonies batchwise. Control experiments showed that our detection limit for deletants was one knockout allele for every 100–500 wild-type alleles (data not shown). For all of the *tbp *and *tfb *genes for which we did not obtain knockouts by the initial screens (*tbp*ABE and *tfb*BDEF), bands corresponding only to the wild-type alleles, but not the deletion alleles were observed. This indicated that these genes were not deleted, even at a frequency of 0.1%, which is consistent with the requirement of these genes for cell viability under standard laboratory growth conditions (Figures 1 and 2).

### Microarray analysis

**Figure 3:**
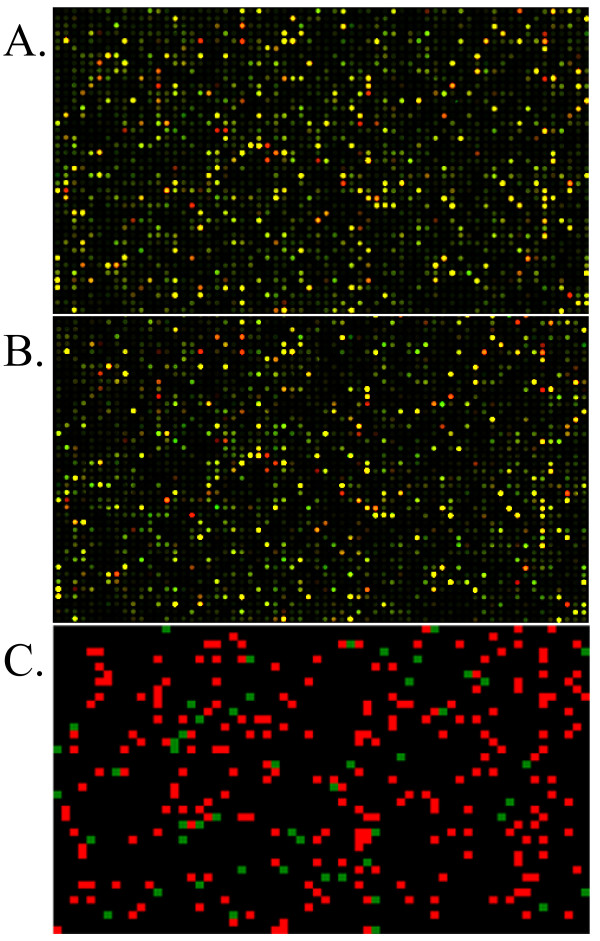
Microarray images. Identical regions of one of the Δ*tbp*D and Δ*tfb*A microarrays are shown (panels A and B, respectively). C. Grid representing the probes that have a fold change value greater than or equal to 1.5 in all four microarrays performed (both biological and technical replicates for Δ*tbp*D and Δ*tfb*A).

**Figure 4:**
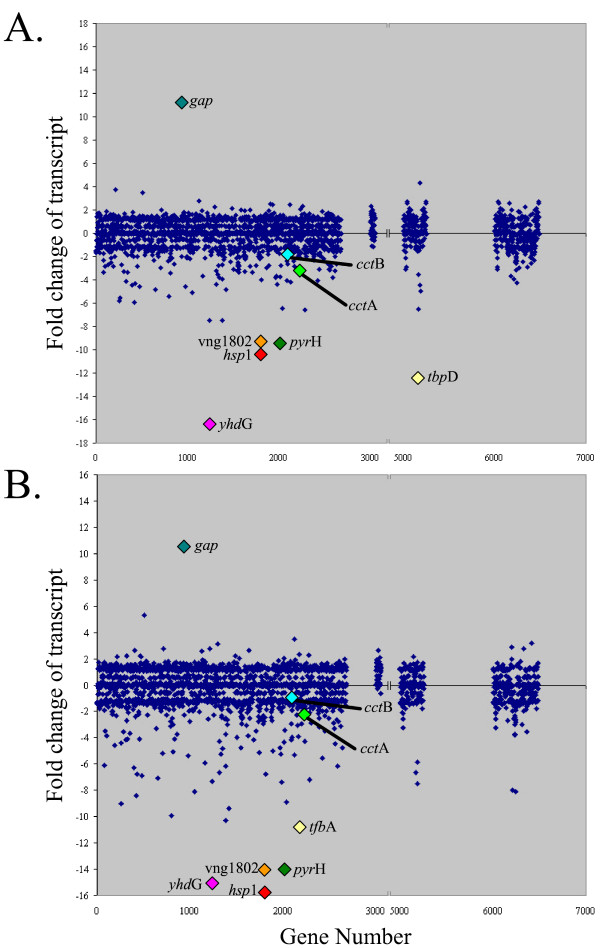
Scatter plot of microarray data from transcription factor knockouts. (A) A plot of the fold-change of each gene in the Δ*tbp*D microarrays. The most up and down regulated genes and some known heat shock response genes are highlighted. (B) A plot of the fold-change of each gene in the Δ*tfb*A microarrays. The most up and down regulated genes and some known heat shock response genes are highlighted.

To test the hypothesis of a novel regulatory system requiring the recognition of different promoters by specific TBP-TFB combinations operating in *Halobacterium *sp. NRC-1, we compared the transcriptomes of two mutants, Δ*tbp*D and Δ*tfb*A (see additional files 1 and 2). We used custom DNA microarrays fabricated using Agilent inkjet technology with oligonucleotides designed with the program OligoPicker. The arrays contained 10,807 60-mer nucleotide features representing 2,677 open reading frames (ORFs) [41,42]. Each knockout mutant was compared to the parent Δ*ura*3 strain and both biological and technical replicates were performed. A significant change in transcript abundance was defined as at least 1.5-fold change (Figures 3 and 4).

**Figure 5:**
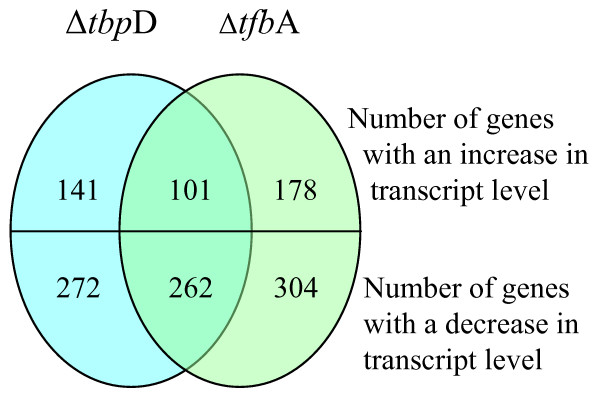
Co-regulation of genes. The figure shows a Venn diagram representing the number of genes regulated in the microarrays for each knockout strain and the number of genes regulated in both strains. The left side represents the Δ*tbp*D arrays and the right side represents the Δ*tfb*A arrays.

Of the 2,677 ORFs on the array, representing 99.9% of the *Halobacterium *sp. NRC-1 genes, 272 (10%) showed lower transcript levels and 141 (5.3%) showed higher transcript levels in the Δ*tbp*D strain while 304 (11.4%) showed lower transcript levels and 178 (6.6%) showed higher transcript levels in the Δ*tfb*A strain (see additional files 1 and 2). Strikingly, a large fraction of the transcriptionally affected genes, 262 genes with less transcript and 101 with more transcript levels were changed by 1.5-fold or greater magnitude in both knockout strains. A total of 96.3 and 86.2% of the genes found to have lower transcript levels in either the Δ*tbp*D and Δ*tfb*A strains, respectively, corresponded to lower transcript levels in the other. This similar depression in transcript level of a large number of genes suggests that the great majority of genes requiring the TbpD factor for transcription also requires the TfbA factor, and vice versa (Figures 3, 4, 5, and Table 2).

**Table 2:** Groups of regulated genes in the Δ*tbpD *and Δ*tfb*A microarrays

	Increased in		Increased in both	Reduced in		Reduced in both
	Δ*tbp*D	Δ*tfb*A		Δ*tbp*D	Δ*tfb*A	
Amino acid metabolism	3	5	1	18	22	18
Carbohydrate and lipid metabolism	5	4	3	13	19	16
Cellular process	10	18	11	39	44	37
Cofactor and secondary metabolites	5	8	4	25	25	23
DNA metabolism	7	10	3	5	12	6
Energy metabolism	6	10	6	16	19	16
General function prediction only	0	8	1	7	4	7
Nucleotide metabolism	5	3	4	6	7	6
RNA	13	11	8	0	0	0
Transcription and regulation	6	17	9	16	9	11
Translation	7	23	10	6	6	5
Transport	7	4	4	17	24	18
Unknown	67	57	37	104	113	99
Total	141	178	101	272	304	262

Genes where expression was changed by 1.5-fold or greater in either the Δ*tbp*D or Δ*tfb*A mutants were grouped into one of 13 functional categories (Table 2). In all cases, the largest groups were the unknowns, accounting for 30 to 45% of all regulated genes. Interestingly, the percentage of genes co-regulated on each replicon is roughly the same for the Δ*tbp*D and Δ*tfb*A microarray. For the genes whose transcripts are decreased in both mutant strains, 85% are on the chromosome, 3% are on pNRC100, and 11% are on pNRC200. For genes whose transcripts are increased in the mutant strains, 87% are on the chromosome, 6% are on pNRC100, 6% are on pNRC200. This suggests that TbpD, whose gene is on pNRC100, is able to pair with TfbA, whose gene in on the chromosome, to coordinate regulation of genes all over the genome but predominately in the chromosome. The location of the regulated genes was checked and found to be distributed essentially randomly over the entire genome.

As expected, the expression of the Δ*tbp*D or Δ*tfb*A mutants was significantly reduced compared to the parent strain, 12.4-fold and 10.8-fold respectively (Table 3). Further, *tfb*A was significantly down regulated (1.5-fold) in the Δ*tbp*D strain. Transcription of the other *tbp *and *tfb *genes was not significantly changed, except *tfb*F in the Δ*tbp*D and Δ*tfb*A strains and *tbp*F in the Δ*tfb*A strain. Further, the transcripts of several important enzymes such as triosephosphate isomerase (*tpi*A), chitinases (*chi *and *chi*2), chemotaxis proteins (*che*C2), gas vesicle gene cluster (*gvp*A-L), small heat shock protein (*hsp*1), and the thermosome (*cct*A) are all down regulated in both mutants (see additional files 1, 2 and 5).

**Table 3:** Fold change in the *tbp *and *tfb *genes in the Δ*tbpD *and Δ*tfb*A microarrays

	Fold Change^a^				Fold Change^a^		
					
Gene Name	Δ*tbp*D microarrays	Δ*tfb*A microarrays	Avg of all 8 microarrays	GeneName	Δ*tbp*D microarrays	Δ*tfb*A microarrays	Avg of all 8 microarrays
*tbpA*	1.166	0.100	0.633	*tfbA*	-1.537	-10.766	-6.152
*tbpB*	0.016	-0.187	-0.085	*tfbB*	-0.024	1.433	0.704
*tbpC*	1.049	0.085	0.567	*tfbC*	1.161	0.000	0.581
*tbpD*	-12.433	-0.591	-6.512	*tfbD*	0.125	-0.027	0.049
*tbpE*	-0.514	0.103	-0.205	*tfbE*	-1.160	-1.345	-1.252
*tbpF*	-1.590	1.143	-0.224	*tfbF*	1.687	2.191	1.939
				*tfbG*	0.110	1.181	0.645

^a^Negative values denote a reduction in transcript levels in the knockout strain

Analysis of the microarray data showed transcriptional changes in several gene clusters, indicating that they are responsive to TbpD and TfbA: vng1–3, vng80–84 (*moe *genes), vng161–162 (glutamate dehydrogenase and medium-chain acyl-CoA ligase), vng811–813 (spermidine/putrescine-binding protein), vng814–817 (quinine oxidoreductase and chitinases), vng1238–1240 (amino acid transporter *yhd*G), vng1314–1315, vng1340–1342 (3-oxoacyl-reductase and N5, N10-methylenetretrahydromethanopterin reductases), vng1550–1566 (cobalamin biosynthesis), vng1632-1365 (cobalt transport), vng1801–1802 (*hsp*1), vng1972–1973 (chains of glycerol-3-phosphate dehydrogenase), vng2217–2220 (pyruvate dehydrogenase, dihydrolipoamide S-acetyltransferase, dihydrolipoamide dehydrogenase, prephenate dehydratase), vng2377–2378 (nitrite/nitrate reduction and copper transport), vng5028–5033 (gas vesicle cluster A), vng6229–6247 (gas vesicle cluster B).

### Heat shock response

It was evident from the microarray data that the gene for one small heat shock protein (*hsp*1) and the thermosome (*cct*A) had reduced transcript levels in both the Δ*tbp*D and Δ*tfb*A mutant strains (-10.3 and -3.1-fold respectively for Δ*tbp*D and -15.8 and -2.2-fold respectively for Δ*tfb*A). Since a reduction in small heat shock proteins has been previously shown to increase the amount of aggregated cytosolic protein [44], we hypothesized that both strains (Δ*tbp*D and Δ*tfb*A) would have slower growth rates at elevated temperatures (i.e. above 42°C). Doubling times were measured for the parent, Δ*tbp*D, and Δ*tfb*A strains to gauge the growth effects at elevated temperatures. Growth curves at 37°C, calculated from the line of least squares for the growth of each strain, indicated that all three strains had doubling times of 13 to 13.5 hours (data not shown). However, growth curves at 49°C showed that the Δ*tbp*D and Δ*tfb*A strains grew more slowly than the Δ*ura*3 strain. Doubling times were 14.4 ± 0.5 for Δ*ura*3, 16.5 ± 0.7 for Δ*tbp*D and 17.3 ± 1.1 hours for Δ*tfb*A (data not shown).

To further confirm the heat sensitive phenotype of both strains, we tested the ability of the Δ*tbp*D and Δ*tfb*A strains to survive at elevated temperatures. Incubating the Δ*ura*3 strain for one hour at a sublethal temperature, 49°C, before exposure to a lethal temperature, 56°C, resulted in protection by a classic heat shock response that could be observed after about two hours, with greater survival (89% survival) compared to direct exposure to 56°C (62% survival) [45]. In contrast, the Δ*tbp*D and Δ*tfb*A strains showed little or no protection from preincubation at 49°C versus direct exposure to 56°C (46 versus 51% for Δ*tbp*D and 58 versus 67% for Δ*tfb*A). These experiments corroborated the results from analysis of growth rates, confirming that the Δ*tbp*D and Δ*tfb*A strains have a reduced ability to grow and survive at higher temperatures (Table 4). The observed reduction in growth rate and decrease in survivability at increased temperatures in the mutants is consistent with a role for the TbpD and TfbA transcription factors in protection of cells to elevated temperature.

**Table 4:** Percent survivability at elevated temperatures

	Δ*ura*3			Δ*tbp*D			Δ*tfb*A		
			
Time	42°C	49 to 56°C	56°C	42°C	49 to 56°C	56°C	42°C	49 to 56°C	56°C
0	100	100	100	100	100	100	100	100	100
1	98.6	94.0	71.7	93.1	86.1	68.1	103.2	72.6	79.4
2	105.9	89.5	61.7	105.6	46.1	51.4	98.4	58.1	67.4
3	99.0	61.9	47.6	104.2	30.0	6.7	103.9	47.4	33.9
4	105.2	38.1	28.6	83.3	2.5	0.8	84.2	16.1	3.5

### Phylogenetic analysis of TBPs and TFBs

**Figure 6:**
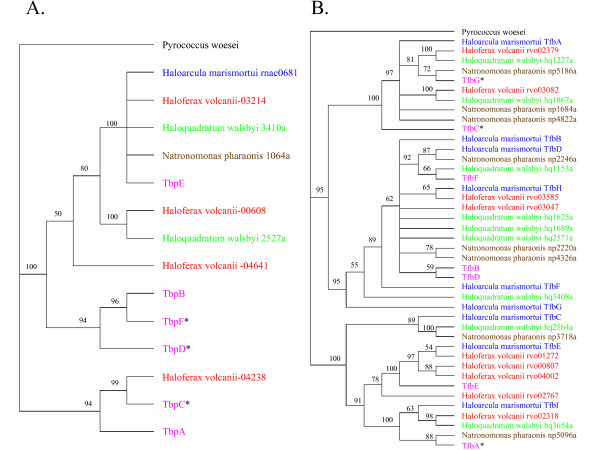
Phylogenetic neighbor-joining trees of the haloarchaeal TBP (A) and TFB (B) protein sequences, with the *Pyrococcus woesei *TBP or TFB sequence used as an outgroup. Asterisks indicate genes that have been knocked out in NRC-1. TBP or TFB genes from *Halobacterium *sp. NRC-1 (pink), *Haloarcula marismortui *(blue), *Haloferax volcanii *(red), *Haloquadratum walsbyi *(green), *Natronomonas pharaonis *(brown), and *Pyrococcus woesei *(black) are shown.

In order to examine the evolutionary relationships of the haloarchaeal TBP and TFB proteins, sequences from *Halobacterium *sp. NRC-1, *Haloarcula marismortui*, *Haloferax volcanii*, *Haloquadratum walsbyi*, *Natronomonas pharaonis*, and *Pyrococcus woesei*, as an outgroup, were used to generate alignments using the ClustalX program and produce phylogenetic trees with the core regions using the PAUP* program. The resulting trees are shown in Figure 6. For the TBP tree, the largest family consists of TbpE from *Halobacterium *sp. NRC-1 and a single member from each of the other four haloarchaea, including the sole TBP from *H. marismortui *and *N. pharaonis*. The other families contained up to three homologs of the canonical TBP protein, with representatives from NRC-1 in two clades, with at least one member being dispensable by gene deletion (asterisks in Figure 6A).

The situation was significantly more complex for TFB proteins, which were present in greater copy numbers (7–9) in all five haloarchaea (Figure 6B). The largest family of TFBs contained three members from *Halobacterium *sp. NRC-1, which did not show knockouts (TfbB, TfbD, and TfbF), and between two and five for each of the other haloarchaea. Another large family contained two members from *Halobacterium *sp. NRC-1 (TfbC and TfbG), both of which were found to be dispensable by knockouts, but each of the other haloarchaea also contained one or more members. A third family contained one member from each of the five haloarchaea, including TfbA from *Halobacterium *sp. NRC-1 and a fourth family contained TfbE from *Halobacterium *sp. NRC-1, for which we were unable to isolate a knockout, and *H. volcanii *(four members) and *H. marismortui *(one member) homologs.

## Discussion

The present study describes a combination of genetic, transcriptomic, and phylogenetic analyses of *tbp *and *tfb *genes and proteins in haloarchaea. Our knockout analysis of *tbp*D and *tfb*A transcription factor genes of *Halobacterium *sp. NRC-1 showed that TbpD regulates 15%, TfbA regulates 18%, and TbpD and TfbA together regulate over 10% of the genes in the NRC-1 genome. These genetic results strongly support the prediction of a novel mechanism of gene regulation where specific TBP-TFB pairs are used for transcription of specific subsets of genes. Our finding that two key heat shock genes, *hsp*1 and *cct*A, are under transcriptional control of TbpD and TfbA factors, and that both the Δ*tbp*D and Δ*tfb*A mutants are sensitive to elevated temperatures, suggest that these factors regulate expression of genes important for survival at increased temperature in this haloarchaeon.

The finding of multiple *tbp *and *tfb *genes in *Halobacterium *sp. NRC-1, a larger total number than for any other archaea or eukaryote, and their involvement in transcription of specific genes is a novel finding [12,14]. These genes are generally found in one or two copies and their use as general transcription factors in most other archaea and eukaryotes is underscored by the fact that the eukaryotic factors can substitute for archaeal factors in vitro [46]. For haloarchaea, we previously predicted that with six TBP factors and seven TFB factors, up to 42 different TBP-TFB combinations may occur [10]. Our results, showing that the knockouts of *tbp*D and *tfb*A (Figure 1) each significantly alter the expression of nearly the same set of genes in the *Halobacterium *genome (Table 2 and additional files 1 and 2), suggests that some factors may have only a few or even only one cognate partner, and the complexity may be considerably lower than we originally hypothesized. However, this may be masked by the fact that a TBP and TFB pair must be involved in the transcription of other TBPs, TFBs, and regulators, the latter of which would result in indirect effects. The requirement of specific partners may also explain why in early studies of transcription in vitro, a purified RNA polymerase of *Halobacterium *sp. did not produce properly initiated transcripts [5,47].

Two of the most highly affected genes in the Δ*tbp*D and Δ*tfb*A mutants were the *hsp*1 and *cct*A genes (Figure 4 and additional files 1, 2 and 5). The *hsp*1 gene is a member of the *hsp*26/42 clade (COG0071), which is a part of the diverse α-crystallin protein family existing in most but not all bacteria, eukaryotes, and archaea and responsible for preventing the non-specific aggregation of proteins [48,49]. Analysis of the genomic sequence of NRC-1 showed that the *hsp*1 gene may be part of a co-ordinately regulated operon whose other member (VNG1802) has no homology with any previously characterized genes, suggesting a novel approach or new member of the response to elevated temperature. The *cct*A gene is homologous to the *gro*EL/*hsp*60 family of proteins (COG0459), a well characterized family of chaperones which have been shown to provide kinetic assistance to polypeptide folding in most bacteria and some archaea [44,50,51]. The other portion of the thermosome in *Halobacterium *sp. NRC-1, *cct*B, and unlinked gene, was also down regulated but just below (-1.43 fold) our 1.5 fold cut-off. The decreased levels of transcript for both these genes coupled with the slower doubling times for both mutant strains strongly suggest that the TbpD and TfbA genes act together to regulate at least a portion of the heat shock response in *Halobacterium *sp. NRC-1.

Previous heat shock studies in the haloarchaea had shown that certain Tfb proteins [16] and small heat shock proteins [52] may be induced at higher temperatures. The *tfb*2 gene of *H*. *volcanii *showed up to an eight-fold increase in transcript levels at elevated temperatures by Northern analysis and the corresponding protein was also shown to increase two-fold by Western analysis after incubation at elevated temperatures [16]. The *tfb*1 gene also showed a slight increase in transcript levels, but an increase in protein could not be confirmed. A more recent proteomic analysis of *Halobacterium *sp. NRC-1 showed that there was an increased abundance of three of the four small heat shock proteins, including the *hsp*1 gene product, and the DnaJ and GrpE homologs under increased temperature. However, changes in TBPs or TFBs were not found in this study [45].

Our inability to knockout seven out of thirteen *tbp *and *tfb *genes (Figures 1 and 2) indicated that many of these genes are probably essential for viability of cells. However, of the five genera for which genome sequences are available, two contained only a single TBP, *H. marismortui*, and *N. pharaonis*, while the other three contained a plurality: six for NRC-1, four for *H. volcanii*, and two for *H. walsbyi*. Not surprisingly, three of the six TBP genes in NRC-1 could be easily knocked out. This suggests that there is likely to be overlapping activity in the function of TBP genes in those haloarchaea which contain multiple genes, or that some TBPs may be required for only a small number of non-essential genes. However, our inability to isolate mutants of three TBP genes (Figure 2), even screening populations of 1,000 or more, is consistent with the hypothesis that several distinct classes of promoters do exist. Our finding of a large number of affected genes in the *tbp*D deletion mutant is consistent with this suggestion. It is tempting to speculate that the plurality of TBP-like proteins in *Halobacterium *may be analogous to that found in some animal systems, where recognition of alternate promoters has been implicated in development [22,53].

In contrast to the relatively small numbers of TBP genes, larger numbers of TFB genes are present across all five genera of haloarchaea, suggesting a more complex function for TFB transcription factors. There may be different classes of promoters which use the same TBP but distinct TFB proteins in promoter recognition. In our prior mutagenic analysis of the *bop *promoter, we found no conservation in the BRE region immediately 5' to the putative TATA-box where the TBP is expected to bind, suggesting the possibility of more variation in these factors in haloarchaea than has been observed in other systems. Like TBPs, our ability to knockout three TFB genes (Figures 1 and 2) indicates that there may be overlap in promoter recognition among some of these proteins. However, it is also possible for TFBs that some members are required only for transcription of a small number of specialized genes, which are not required under standard laboratory growth conditions. Interestingly, in a recent report, either one of two closely related TFBs genes in the thermophilic archaeon, *Thermococcus kodakaraensis*, may be deleted individually but not together. This suggests that only those organisms with divergent TFBs and TBPs, like *Halobacterium *sp. NRC-1, can use the transcription factors for promoter selection [54].

Another recent report [55] covering the essentiality of TBPs and TFBs in *Halobacterium *suggests that *tbp*ACD and *tfb*ABCDE are not essential for growth under standard laboratory conditions. The control assays used for our PCR-based mutant screens suggested that we could detect a knockout allele at a frequency of 0.1% in a population. This leaves open the possibility that we were unable to identify knockouts present at low frequency. However, it is difficult to compare the results directly, since no primary data is provided and a differing method to create the knockouts was used. Further, the authors of the recent report grew their cultures at 37°C, not 42°C as we have. Thus, it is possible that some of the discrepancies in essentiality arose from this difference in growth temperature. This makes sense in light of our heat shock data where a one-hour incubation at a sublethal temperature (49°C) before exposure to a lethal temperature (56°C) resulted in greater survival. This response seems to be regulated at least partially by *tbp*D and *tfb*A; however, it may also require other basal transcription factors that are unnecessary at lower temperatures. Finally, the authors did not observe a correlation between *tbp*D and *tfb*A in their ChIP-Chip analysis or evolutionary relationship analysis of multiple microarrays covering seven different experimental variables. This may be due to the shortcomings of their ChIP-Chip approach as a majority of the genes they surveyed showed no interaction with any basal transcription factor and the fact that it is difficult to tweeze out fine details such as basal transcription interaction in multiple microarrays performed under varying conditions.

Our results provide the first in-depth analysis of the multiple TBP and TFB factors in the model haloarchaeon, *Halobacterium *sp. NRC-1. We find that a pair of factors, TbpD and TfbA, is required for coordinate transcription of over 10% of the genome of *Halobacterium *sp. NRC-1. This remarkable finding confirms one of the predictions of earlier studies suggesting that specific TBP-TFB pairs may be required for transcription of certain genes in haloarchaea [15] and therefore are involved in a novel mechanism of gene regulation in the third Domain of life. We speculate that this mechanism may have evolved to handle the great environmental dynamics experienced in hypersaline environments. The finding of reduction of growth rate at 49°C and a decrease in survival at 56°C in the absence of TbpD and TfbA is consistent with a critical biological function.

## Methods

### Strains, culturing, and construction of knockouts

*Escherichia coli *strain DH5α was used for construction and propagation of plasmids. Plasmid pBB400 or pMPK408, containing the *ura*3 and *bla *genes, was used as the vector for construction of gene knockouts [26]. Knockout constructs were created by first cloning, into the *Sma*I site, a PCR amplified fragment containing the coding region of the target gene and ~500 bp of sequence upstream and downstream (see additional file 3). For construction of gene deletion plasmids, inverse primer pairs hybridizing near the start and stop codons of the target genes, including several codons, were used for PCR amplification and the linear product was ligated to circularize (see additional file 4).

A uracil auxotroph *Halobacterium *strain (Δ*ura*3) was used as the host for construction of the *tbp *and *tfb *knockouts [31]. The Δ*ura*3 strain was grown in CM^+ ^media supplemented with 5-FOA and competent cells were obtained through the addition of EDTA and PEG, as previously described [24]. After transformation with knockout plasmids, integrants were selected on agar plates containing uracil drop-out media. DNA was purified from individual colonies and integrant strains confirmed by PCR amplification of the *bla *gene. Excisants were selected on CM^+ ^media with 0.25 mg/ml 5-FOA. DNA was purified from either individual colonies or by collecting ~1,000 colonies from a CM^+^-5-FOA plate and screened by PCR amplification using upstream and downstream primers [31,42] (see additional files 3 and 4).

### Whole genome microarray analysis

The Δ*ura*3, Δ*ura*3Δ*tbp*D, and Δ*ura*3Δ*tfb*A *Halobacterium *strains were aerobically grown in batch cultures, shaken in a New Brunswick G25 rotary shaker at 220 rpm, to exponential phase (OD_600 _= 1) at 42°C. Before harvesting the cells, cultures were swirled in an ice-water bath to rapidly cool the cultures and "freeze" the RNA profile. Total RNA was isolated using the Agilent total RNA isolation mini kit (Agilent Technologies, Palo Alto, CA) and then treated with DNase. cDNA was prepared with equal amounts of RNA from control and experimental samples and then fluorescently labeled with *Cy*3-dCTP and *Cy*5-d-CTP. Concentrations of RNA and cDNA were measured using a Nanodrop (ND-1000 spectrophotometer). Incorporation of the *Cy*-3 and *Cy*-5 labels was checked via gel electrophoresis and scanning on a GE Typhoon fluorescence scanner. Both biological replicates (independent cultures) and technical replicates (dye swap) were performed for each paring (i.e. knockout and Δ*ura*3 strain) [42]. The same Δ*ura*3 culture was used as the control in all arrays. Washing and hybridization of the arrays was performed as recommended by Agilent and previously described [41]. Slides were scanned for *Cy*-3 and *Cy*-5 signals with an Agilent DNA-microarray scanner.

Oligonucleotide arrays, in situ synthesized using ink-jet technology, were used for transcriptome analysis of the strains [56]. Oligomer (60-mer) probes have been designed for 2,677 (99.9%) ORFs utilizing OligoPicker [57]. These microarrays were thoroughly tested for linearity of response and statistical significance in related studies of *Halobacterium *sp. NRC-1 [41,42]. Signal intensities with a dynamic range in excess of three orders of magnitude were found allowing simultaneous analysis of low- and high-intensity features. Probe signals were extracted and initial analysis was done with the Agilent Feature Extraction Software, where signal from each channel was normalized using the LOWESS algorithm [58] to remove intensity-dependent effects within the calculated values. The data was also log_2 _transformed and parsed using an Excel script [43]. Genes showing greater than 1.5 fold change in transcript abundance in at least two of the replicates with an illuminant intensity greater than 7 and less than 14 were selected for further analysis [59].

### Phenotypic analysis

Growth curves were measured for 25 ml cultures grown in CM^+ ^in Klett flasks, starting with an initial Klett reading of 20, grown at either 37 or 49°C in an Innova 4230 rotary shaker shaken at 220 rpm. Measurements were taken for four cultures at 37°C and eight cultures at 49°C per knockout until late log phase on a Klett-Summerson Photoelectric Colorimeter and doubling times derived by calculating the line of least squares from 20–45 hours of growth post inoculation (exponential phase). The effects of heat killing were also measured for each strain. For this, cultures were grown at 42°C and then incubated at 49°C for 1 hour then shifted to 56°C for 2 hours or 56°C for 3 hours. Aliquots were taken from cultures at all temperatures at one hour intervals and plated onto CM^+ ^media and allowed to grow at 37°C.

### Phylogenetic analysis

The amino acid sequences of the TBPs and TFBs from *Halobacterium *sp. NRC-1 (AAC82815, AAC82955, AAC82884, AAC82898, NP_280885, NP_395899, NP_280839, NP_279732, NP_395840, NP_279833, NP_395867, NP_279414, NP_279370), *Haloarcula marismortui *(YP_135388, YP_135994, YP_138046, YP_136462, YP_134372, YP_136614, YP_134329, YP_135787, YP_137236), *Haloferax volcanii *(http://archaea.ucsc.edu [UCSC archaeal genome browser]), *Haloquadratum walsbyi *(CAJ53507, CAJ52639, CAJ53741, CAJ53505, CAJ52683, CAJ52676, CAJ51995, CAJ51817, CAJ51753, CAJ51356, CAJ51283), *Natronomonas pharaonis *(YP_326192, YP_326499, YP_326761, YP_326774, YP_327502, YP_330892, YP_331136, YP_331271, YP_331316), and *Pyrococcus woesei *(U10285, P61999) were downloaded from NCBI and sequences were aligned with ClustalX (Version 1.81) for Windows XP. The core regions of the molecules were used in distance analysis with the neighbor-joining algorithm, with 10,000 bootstrap replicates, performed using PAUP* in the GCG Wisconsin Package (version 10) running on a Silicon Graphics workstation. Core regions were defined as those residues aligning with amino acids 3–293 of the TBP and 101–300 of the TFB from *P. woesei*.

## Authors' contributions

JAC performed research and drafted the manuscript. SD supervised the research, including design, data analysis, and finalized the manuscript. All authors have read and approved the final manuscript.

## Supplementary Material

Additional file 1 Average values for all genes in the two Δ*tbp*D microarrays and two dye swapped Δ*tbp*D microarrays. Average values for log_2_(x), standard deviation of log_2_(x), fold changes, and p value for the two Δ*tbp*D and the two Δ*tbp*D dye swap arrays. Click here for file

Additional file 2 Average values for all genes in the two Δ*tfb*A microarrays and two dye swapped Δ*tfb*A microarrays. Average values for log_2_(x), standard deviation of log_2_(x), fold changes, and p value for the two Δ*tfb*A and the two Δ*tfb*A dye swap arrays. Click here for file

Additional file 3 List of primers used in creating the first constructs. List of primers used to amplify the *tbp*/*tfb *genes and their surrounding area to create the first constructs and screen for knockouts. Click here for file

Additional file 4 List of primers used in creating the knockout constructs. List of primers used to amplify most of the first constructs to create the *tbp*/*tfb *knockout constructs used in this study. Click here for file

Additional file 5 List of the microarray data of the heat shock response genes from *Halobacterium *sp. NRC-1. Average values for log_2_(x), standard deviation of log_2_(x), fold changes, and p value for the heat shock gene transcripts in the two Δ*tbp*D and the two Δ*tbp*D dye swap arrays and the two Δ*tfb*A and the two Δ*tfb*A dye swap arrays. Click here for file

## References

[B1] Bell SD, Jackson SP (1998). Transcription and translation in Archaea: a mosaic of eukaryal and bacterial features. Trends Microbiol.

[B2] Bell SD, Jackson SP (2000). Mechanism of autoregulation by an archaeal transcriptional repressor. J Biol Chem.

[B3] Reich CI, McNeil LK, Brace JL, Brucker JK, Olsen GJ (2001). Archaeal RecA homologues: different response to DNA-damaging agents in mesophilic and thermophilic Archaea. Extremophiles.

[B4] Thomm M, Wich G (1988). An archaebacterial promoter element for stable RNA genes with homology to the TATA box of higher eukaryotes. Nucleic Acids Res.

[B5] Zillig W, Stetter KO, Tobien M (1978). DNA-dependent RNA polymerase from Halobacterium halobium. Eur J Biochem.

[B6] Littlefield O, Korkhin Y, Sigler PB (1999). The structural basis for the oriented assembly of a TBP/TFB/promoter complex. Proc Natl Acad Sci U S A.

[B7] Ouhammouch M, Geiduschek EP (2005). An expanding family of archaeal transcriptional activators. Proc Natl Acad Sci U S A.

[B8] Reiter WD, Hudepohl U, Zillig W (1990). Mutational analysis of an archaebacterial promoter: essential role of a TATA box for transcription efficiency and start-site selection in vitro. Proc Natl Acad Sci U S A.

[B9] Goede B, Naji S, von Kampen O, Ilg K, Thomm M (2006). Protein-protein interactions in the archaeal transcriptional machinery: binding studies of isolated RNA polymerase subunits and transcription factors. J Biol Chem.

[B10] Baliga NS, DasSarma S (2000). Saturation mutagenesis of the haloarchaeal bop gene promoter: identification of DNA supercoiling sensitivity sites and absence of TFB recognition element and UAS enhancer activity. Mol Microbiol.

[B11] Baliga NS, DasSarma S (1999). Saturation mutagenesis of the TATA box and upstream activator sequence in the haloarchaeal bop gene promoter. J Bacteriol.

[B12] Ng WV, Kennedy SP, Mahairas GG, Berquist B, Pan M, Shukla HD, Lasky SR, Baliga NS, Thorsson V, Sbrogna J, Swartzell S, Weir D, Hall J, Dahl TA, Welti R, Goo YA, Leithauser B, Keller K, Cruz R, Danson MJ, Hough DW, Maddocks DG, Jablonski PE, Krebs MP, Angevine CM, Dale H, Isenbarger TA, Peck RF, Pohlschroder M, Spudich JL, Jung KW, Alam M, Freitas T, Hou S, Daniels CJ, Dennis PP, Omer AD, Ebhardt H, Lowe TM, Liang P, Riley M, Hood L, DasSarma S (2000). Genome sequence of Halobacterium species NRC-1. Proc Natl Acad Sci USA.

[B13] Leffers H, Gropp F, Lottspeich F, Zillig W, Garrett RA (1989). Sequence, organization, transcription and evolution of RNA polymerase subunit genes from the archaebacterial extreme halophiles Halobacterium halobium and Halococcus morrhuae. J Mol Biol.

[B14] DasSarma S, Fraser T, Read T and Nelson KE (2004). Genome sequence of an extremely halophilic archaeon. Microbial Genomes.

[B15] Baliga NS, Goo YA, Ng WV, Hood L, Daniels CJ, DasSarma S (2000). Is gene expression in Halobacterium NRC-1 regulated by multiple TBP and TFB transcription factors?. Mol Microbiol.

[B16] Thompson DK, Palmer JR, Daniels CJ (1999). Expression and heat-responsive regulation of a TFIIB homologue from the archaeon Haloferax volcanii. Mol Microbiol.

[B17] Danner S, Soppa J (1996). Characterization of the distal promoter element of halobacteria in vivo using saturation mutagenesis and selection. Mol Microbiol.

[B18] Baliga NS, Kennedy SP, Ng WV, Hood L, DasSarma S (2001). Genomic and genetic dissection of an archaeal regulon. Proc Natl Acad Sci U S A.

[B19] Berk AJ (2000). TBP-like factors come into focus. Cell.

[B20] Faiger H, Ivanchenko M, Cohen I, Haran TE (2006). TBP flanking sequences: asymmetry of binding, long-range effects and consensus sequences. Nucleic Acids Res.

[B21] Hansen SK, Takada S, Jacobson RH, Lis JT, Tjian R (1997). Transcription properties of a cell type-specific TATA-binding protein, TRF. Cell.

[B22] Holmes MC, Tjian R (2000). Promoter-selective properties of the TBP-related factor TRF1. Science.

[B23] DasSarma S, Berquist BR, Coker JA, DasSarma P, Muller JA (2006). Post-genomics of the model haloarchaeon Halobacterium sp. NRC-1. Saline Systems.

[B24] DasSarma S, DasSarma S and Fleischmann EM (1995). Halophilic Archaea. Halophiles.

[B25] DasSarma S, DasSarma P (2006). Halophiles. Encyclopedia of Life Sciences.

[B26] Berquist BR, Muller JA, DasSarma P, DasSarma S, Oren A and Rainey F (2006). Genetic systems for halophilic archaea. Methods in Microbiology.

[B27] Bergqvist S, Williams MA, O'Brien R, Ladbury JE (2003). Halophilic adaptation of protein-DNA interactions. Biochem Soc Trans.

[B28] Ng WL, DasSarma S (1993). Minimal replication origin of the 200-kilobase Halobacterium plasmid pNRC100. J Bacteriol.

[B29] Cline SW, Lam WL, Charlebois RL, Schalkwyk LC, Doolittle WF (1989). Transformation methods for halophilic archaebacteria. Can J Microbiol.

[B30] Brochier C, Forterre P, Gribaldo S (2004). Archaeal phylogeny based on proteins of the transcription and translation machineries: tackling the Methanopyrus kandleri paradox. Genome Biol.

[B31] Peck RF, DasSarma S, Krebs MP (2000). Homologous gene knockout in the archaeon Halobacterium salinarum with ura3 as a counterselectable marker. Mol Microbiol.

[B32] Peck RF, Echavarri-Erasun C, Johnson EA, Ng WV, Kennedy SP, Hood L, DasSarma S, Krebs MP (2001). brp and blh are required for synthesis of the retinal cofactor of bacteriorhodopsin in Halobacterium salinarum. J Biol Chem.

[B33] Peck RF, Johnson EA, Krebs MP (2002). Identification of a lycopene beta-cyclase required for bacteriorhodopsin biogenesis in the archaeon Halobacterium salinarum. J Bacteriol.

[B34] Woodson JD, Peck RF, Krebs MP, Escalante-Semerena JC (2003). The cobY gene of the archaeon Halobacterium sp. strain NRC-1 is required for de novo cobamide synthesis. J Bacteriol.

[B35] Woodson JD, Zayas CL, Escalante-Semerena JC (2003). A new pathway for salvaging the coenzyme B12 precursor cobinamide in archaea requires cobinamide-phosphate synthase (CbiB) enzyme activity. J Bacteriol.

[B36] Woodson JD, Escalante-Semerena JC (2004). CbiZ, an amidohydrolase enzyme required for salvaging the coenzyme B12 precursor cobinamide in archaea. Proc Natl Acad Sci U S A.

[B37] Woodson JD, Reynolds AA, Escalante-Semerena JC (2005). ABC transporter for corrinoids in Halobacterium sp. strain NRC-1. J Bacteriol.

[B38] Wang G, Kennedy SP, Fasiludeen S, Rensing C, DasSarma S (2004). Arsenic resistance in Halobacterium sp. strain NRC-1 examined by using an improved gene knockout system. J Bacteriol.

[B39] Baliga NS, Bjork SJ, Bonneau R, Pan M, Iloanusi C, Kottemann MC, Hood L, DiRuggiero J (2004). Systems level insights into the stress response to UV radiation in the halophilic archaeon Halobacterium NRC-1. Genome Res.

[B40] Baliga NS, Pan M, Goo YA, Yi EC, Goodlett DR, Dimitrov K, Shannon P, Aebersold R, Ng WV, Hood L (2002). Coordinate regulation of energy transduction modules in Halobacterium sp. analyzed by a global systems approach. Proc Natl Acad Sci U S A.

[B41] McCready S, Muller JA, Boubriak I, Berquist BR, Ng WL, DasSarma S (2005). UV irradiation induces homologous recombination genes in the model archaeon, Halobacterium sp. NRC-1. Saline Systems.

[B42] Müller JA, DasSarma S (2005). Genomic analysis of anaerobic respiration in the archaeon Halobacterium sp. strain NRC-1: dimethyl sulfoxide and trimethylamine N-oxide as terminal electron acceptors. J Bacteriol.

[B43] Coker JA, Dassarma P, Kumar J, Müller JA, Dassarma S (2007). Transcriptional profiling of the model Archaeon Halobacterium sp. NRC-1: responses to changes in salinity and temperature. Saline Systems.

[B44] Narberhaus F (2002). Alpha-crystallin-type heat shock proteins: socializing minichaperones in the context of a multichaperone network. Microbiol Mol Biol Rev.

[B45] Shukla HD (2006). Proteomic analysis of acidic chaperones, and stress proteins in extreme halophile Halobacterium NRC-1: a comparative proteomic approach to study heat shock response. Proteome Sci.

[B46] Wettach J, Gohl HP, Tschochner H, Thomm M (1995). Functional interaction of yeast and human TATA-binding proteins with an archaeal RNA polymerase and promoter. Proc Natl Acad Sci U S A.

[B47] Madon J, Zillig W (1983). A form of the DNA-dependent RNA polymerase of Halobacterium halobium, containing an additional component, is able to transcribe native DNA. Eur J Biochem.

[B48] Cashikar AG, Schirmer EC, Hattendorf DA, Glover JR, Ramakrishnan MS, Ware DM, Lindquist SL (2002). Defining a pathway of communication from the C-terminal peptide binding domain to the N-terminal ATPase domain in a AAA protein. Mol Cell.

[B49] Haslbeck M, Walke S, Stromer T, Ehrnsperger M, White HE, Chen S, Saibil HR, Buchner J (1999). Hsp26: a temperature-regulated chaperone. EMBO J.

[B50] Klunker D, Haas B, Hirtreiter A, Figueiredo L, Naylor DJ, Pfeifer G, Muller V, Deppenmeier U, Gottschalk G, Hartl FU, Hayer-Hartl M (2003). Coexistence of group I and group II chaperonins in the archaeon Methanosarcina mazei. J Biol Chem.

[B51] Chapman E, Farr GW, Usaite R, Furtak K, Fenton WA, Chaudhuri TK, Hondorp ER, Matthews RG, Wolf SG, Yates JR, Pypaert M, Horwich AL (2006). Global aggregation of newly translated proteins in an Escherichia coli strain deficient of the chaperonin GroEL. Proc Natl Acad Sci U S A.

[B52] Daniels CJ, McKee AH, Doolittle WF (1984). Archaebacterial heat-shock proteins. EMBO J.

[B53] Bartfai R, Balduf C, Hilton T, Rathmann Y, Hadzhiev Y, Tora L, Orban L, Muller F (2004). TBP2, a vertebrate-specific member of the TBP family, is required in embryonic development of zebrafish. Curr Biol.

[B54] Santangelo TJ, Cubonova L, James CL, Reeve JN (2007). TFB1 or TFB2 is sufficient for Thermococcus kodakaraensis viability and for basal transcription in vitro. J Mol Biol.

[B55] Facciotti MT, Reiss DJ, Pan M, Kaur A, Vuthoori M, Bonneau R, Shannon P, Srivastava A, Donohoe SM, Hood LE, Baliga NS (2007). General transcription factor specified global gene regulation in archaea. Proc Natl Acad Sci U S A.

[B56] Hughes TR, Mao M, Jones AR, Burchard J, Marton MJ, Shannon KW, Lefkowitz SM, Ziman M, Schelter JM, Meyer MR, Kobayashi S, Davis C, Dai H, He YD, Stephaniants SB, Cavet G, Walker WL, West A, Coffey E, Shoemaker DD, Stoughton R, Blanchard AP, Friend SH, Linsley PS (2001). Expression profiling using microarrays fabricated by an ink-jet oligonucleotide synthesizer. Nat Biotechnol.

[B57] Wang X, Seed B (2003). Selection of oligonucleotide probes for protein coding sequences. Bioinformatics.

[B58] Cleveland WS (1979). Robust locally weighted regression and smoothing scatterplots. J Am Stat Assoc.

[B59] Quackenbush J (2002). Microarray data normalization and transformation. Nat Genet.

