# Supplementary material for: Genetic and transcriptomic analysis of transcription factor genes in the model halophilic Archaeon: coordinate action of TbpD and TfbA
Source: BMC Genet. 2007 Sep 24;8:61. doi: 10.1186/1471-2156-8-61 (PMC2121645; doi:10.1186/1471-2156-8-61)
Supplement: Additional file 3 — List of primers used in creating the first constructs. List of primers used to amplify the tbp/tfb genes and their surrounding area to create the first constructs and screen for knockouts. [file 1471-2156-8-61-S3.pdf]

Primers used for creation of first construct

Gene area amplified	Primer Name	5' Primer Sequence	Primer Name	3' Primer Sequence	Resulting Plasmid
*tbp*A	tbpA' Rev	agacggatgctccgatacgta	5038 For	atgaacggtgacgcctcaata	pMPK408-*tbp*A
*tbp*B	5050 For	atgcacgatttaactggatt	5053 For	atgggtgagaactgtgataa	pBB400-*tbp*B
*tbp*C	tbpC' For	ggcatccttttcacgtaccg	tbpC' Rev	caggtgttcacgctccctgt	pMPK408-*tbp*C
*tbp*D	5160 Rev	ggcttcggtctgccaggtac	5164 Rev	tcatagttggcaaggtcgtc	pBB400-*tbp*D
*tbp*E	tbpE' For	aagcagtcgtcggcggtgaa	tbpE' Rev	gtactcgtccatgcgcgtcac	pBB400-*tbp*E
*tbp*F	6437 For	atggagattccatcgcaact	tbpF' Rev	cttcctgagcagcagatgta	pBB400-*tbp*F
*tfb*A	2183 For	atgagggtggcgttcggcga	2185 Rev	gacgccgtccacgcccgcctc	pMPK408-*tfb*A
*tfb*B	733 For	atgtcgccgataccactgcc	735 Rev	tcagtttgcgttggcgccgc	pBB400-*tfb*B
*tfb*C	6349 Rev	ttagtacgtctgtggtggct	6353 For	atgaccaccaaacagttcac	pMPK408-*tfb*C
*tfb*D	868 Rev	ctccccggcggccgcttcga	870 Rev	cgagacgttggggcctttga	pBB400-*tfb*D
*tfb*E	6387 Rev	tcatttcgtatcctcagtat	6391 For	atggctcgaaccaaaatggg	pBB400-*tfb*E
*tfb*F	314 Rev	ctgtaggtcggcgatcagcg	316 Rev	ctaggtggcgtggagctggc	pMPK408-*tfb*F
*tfb*G	255 Rev	cgtcgagggcctcgttggcg	252 Rev	agtgaggagccggacacgca	pBB400-*tfb*G
