# Supplementary material for: Genetic and transcriptomic analysis of transcription factor genes in the model halophilic Archaeon: coordinate action of TbpD and TfbA
Source: BMC Genet. 2007 Sep 24;8:61. doi: 10.1186/1471-2156-8-61 (PMC2121645; doi:10.1186/1471-2156-8-61)
Supplement: Additional file 4 — List of primers used in creating the knockout constructs. List of primers used to amplify most of the first constructs to create the tbp/tfb knockout constructs used in this study. [file 1471-2156-8-61-S4.pdf]

## Primers used for creation of knockout construct

| Primer Name | Primer Position | Primer Sequence | Number of 5' Codons | Primer Name | Primer Position | Primer Sequence | Number of 3' Condons | Resulting Plasmid |
|---|---|---|---|---|---|---|---|---|
| *tbp*A 5' inverse | 5'For | gtccattgcgacccgcgaga | 2 | *tbp*A 3' inverse | 3'Rev | aaataaggtaacgaccgtac | 2 | pSF241 |
| *tbp*B 5' inverse | 5'For | gctcatacggggtattcgtc | 2 | *tbp*B 3' inverse | 3'Rev | cttgactgagcccatggtct | 2 | pJAC2 |
| *tbp*C 5' inverse | 5'For | cgtcatctctctcccggttg | 2 | *tbp*C 3' inverse | 3'Rev | gaattagtttaatcggattt | 2 | pSF251 |
| *tbp*D 5' inverse | 5'For | actcattcggtcaccttgtc | 2 | *tbp*D 3' inverse | 3'Rev | gagtaacccaccacaacagc | 2 | pJAC4 |
| *tbp*E 5' inverse | 5'For | ggtcatcgttcgacttaaac | 2 | *tbp*E 3' inverse | 3'Rev | gactaggcgggcatggtggc | 2 | pJAC5 |
| *tbp*F 5' inverse | 5'For | gctcatacgggtattcgtcg | 2 | *tbp*F 3' inverse | 3'Rev | gagtgagtccacggagctct | 2 | pJAC6 |
| *tfb*A 5' inverse | 5'For | gcctaatcagccaccaccac | 2 | *tfb*A 3' inverse | 3'Rev | actcattatgttggaagtgc | 2 | pSF271 |
| *tfb*B 5' inverse | 5'For | actcattatgttggaagtgc | 2 | *tfb*B 3' inverse | 3'Rev | gcctgacgccgcctgttttc | 2 | pJAC8 |
| *tfb*C 5' inverse | 5'For | ttccattcaccatatgacaa | 2 | *tfb*C 3' inverse | 3'Rev | gtataaactgctaactcggc | 2 | pSF281 |
| *tfb*D 5' inverse | 5'For | tgtcattgtggggctggctg | 2 | *tfb*D 3' inverse | 3'Rev | gcgtaacccatattcggggc | 2 | pJAC9 |
| *tfb*E 5' inverse | 5'For | cgtcatcggtctcacctgat | 2 | *tfb*E 3' inverse | 3'Rev | gaatagagagctcgtatagcc | 2 | pJAC10 |
| *tfb*F 5' inverse | 5'For | tgtcatgtagcgtacagccc | 2 | *tfb*F 3' inverse | 3'Rev | gtgtagacgtcacaccccgc | 2 | pSF291 |
| *tfb*G 5' inverse | 5'For | ggaccgtgtcatcgcatttt | 4 | *tfb*G 3' inverse | 3'Rev | attcacggctgagccctggc | 4 | pJAC11 |
